# Genomic identification of WRKY transcription factors in carrot (*Daucus carota*) and analysis of evolution and homologous groups for plants

**DOI:** 10.1038/srep23101

**Published:** 2016-03-15

**Authors:** Meng-Yao Li, Zhi-Sheng Xu, Chang Tian, Ying Huang, Feng Wang, Ai-Sheng Xiong

**Affiliations:** 1 State Key Laboratory of Crop Genetics and Germplasm Enhancement, College of Horticulture, Nanjing Agricultural University, 1 Weigang, Nanjing 210095, China

## Abstract

WRKY transcription factors belong to one of the largest transcription factor families. These factors possess functions in plant growth and development, signal transduction, and stress response. Here, we identified 95 *DcWRKY* genes in carrot based on the carrot genomic and transcriptomic data, and divided them into three groups. Phylogenetic analysis of WRKY proteins from carrot and *Arabidopsis* divided these proteins into seven subgroups. To elucidate the evolution and distribution of WRKY transcription factors in different species, we constructed a schematic of the phylogenetic tree and compared the WRKY family factors among 22 species, which including plants, slime mold and protozoan. An in-depth study was performed to clarify the homologous factor groups of nine divergent taxa in lower and higher plants. Based on the orthologous factors between carrot and *Arabidopsis*, 38 DcWRKY proteins were calculated to interact with other proteins in the carrot genome. Yeast two-hybrid assay showed that DcWRKY20 can interact with DcMAPK1 and DcMAPK4. The expression patterns of the selected *DcWRKY* genes based on transcriptome data and qRT-PCR suggested that those selected *DcWRKY* genes are involved in root development, biotic and abiotic stress response. This comprehensive analysis provides a basis for investigating the evolution and function of *WRKY* genes.

Transcriptional regulation is the most important link for regulating gene expression in plants. Transcription factors are involved in controlling many important biological processes in the gene transcription regulatory network^1^,^2^. In the plant genome, a large proportion of genes belong to transcription factors, and at least 58 transcription factor families have been determined^3^. In *Arabidopsis* and rice, approximately 8% and 4% of genes were identified as transcription factors, respectively^4^,^5^.

The WRKY family, whose name is derived from the highly conserved WRKY domain, is one of the largest transcription factor families^6^. The WRKY domain contains about 60 amino acids, comprising a highly conserved short peptide WRKYGQK and adjacent C_2_H_2_ or C_2_HC zinc finger structure. The conserved amino acid WRKYGQK also consists of various forms, such as WRKYGKK, WRKYDQK, and WRKYDHK^7^,^8^. Based on the number of WRKY domains and the type of zinc finger, the WRKY family can be divided into three groups, namely, groups I, II, and III. Group I contains two WRKY domains and a C_2_H_2_ zinc finger type (C-X_4–5_-C-X_22–23_-H-X_1_-H). Group II contains one WRKY domain and a C_2_H_2_ type zinc finger; this group can be further divided into five subgroups (IIa, IIb, IIc, IId, and IIe). Group III also contains only one domain but has a C_2_HC zinc finger type (C-X_7_-C-X_23_-H-X_1_-C)^6^. Wu and his colleagues further analyzed the WRKY family in *Arabidopsis* and rice and revealed the distribution of group II into three subgroups (IIa+b, IIc, and IId+e) based on sequence similarities^9^.

The first reported *WRKY* gene, *SPF1*, was cloned from sweet potato in 1994^10^. Since then, several *WRKY* genes have been found in parsley^11^, *Arabidopsis*^12^, rice^13^, and even in green algae^14^. The completed genome sequencing of many plants has resulted in a more comprehensive identification of *WRKY* genes. The number of WRKY members in different species varies greatly. Thus far, 72 members have been found in *Arabidopsis*^15^, 100 in rice^9^, 41 in *Physcomitrella patens*^3^,^16^, and 19 in *Selaginella moellendorffii*^3^. Evolution analysis showed that group I was the oldest group, and groups II and III originated from group I^9^,^17^. In the later study, WRKY factors were identified in the nonplant species *Dictyostelium discoideum* and *Giardia lamblia*, suggesting that WRKY factors have a very ancient origin^14^.

A large number of WRKY factors were found, but the functions of only a small number of these factors have been analyzed further. These WRKY factors are involved in seed germination, plant growth and development, signal transduction, and metabolic regulation^18^,^19^,^20^. They also participate in biotic and abiotic stress responses, such as freezing, salinity, drought, and pathogen infection^21^,^22^. A WRKY transcription factor, ABO3, mediates plant responses to ABA and drought stress in *Arabidopsis*^23^. Overexpression of two wheat *WRKY* genes, namely, *TaWRKY2* and *TaWRKY19*, confer tolerance to salt, drought, and freezing stresses in transgenic plants^24^. Similarly, Zhou *et al.* revealed that three *GmWRKY* genes from soybean play differential roles in abiotic stress tolerance in transgenic *Arabidopsis* plants^25^. Several *WRKY* genes are regulated by miRNAs^26^,^27^. In sunflower, miR396 regulates *HaWRKY6* in response to high-temperature damage^27^.

Carrot is an important economic crop in the family Apiaceae and is rich in carotene and various nutrients. Studies on carrot transcription factors are rarely reported because of the lack of carrot genomic data^28^,^29^. The publication of the carrot genome draft database (CarrotDB) provides resources to make a bioinformatics identification and analysis on WRKY transcription factors. The genomic and transcriptomic database for carrot was built by our group (Lab of Apiaceae Plant Genetics and Germplasm Enhancement, http://apiaceae.njau.edu.cn/carrotdb/index.php)^30^. In this paper, we identified 95 *DcWRKY* genes in the genomic and transcriptomic carrot database and focused on the evolution and duplication of *WRKY* genes on different species. A total of 71 *DcWRKY* genes were detected to have expression on carrot root development based on transcriptome data. Moreover, the expression analysis of several *DcWRKY* genes under different stresses showed that *DcWRKY* genes participated in the abiotic stress response. Our results provide a basis for studying the evolution and function of WRKY transcription factors.

## Results

### Identification of DcWRKY transcription factors in the *D. carota* genome

To identify all the WRKY factors in the carrot genome, we employed the HMM profile of the WRKY domain (PF03106) as a query to search against the database using HMMER3.0 and BLAST. A total of 95 nonredundant genes were assigned as *WRKY* genes and termed *DcWRKY1* to *DcWRKY95* (Table 1 and S1). The lengths of the DcWRKY proteins ranged from 101 to 865 amino acids, with an average of 333 amino acids. The highly conserved domain WRKYGQK was present in 88 DcWRKY proteins, whereas the remaining seven proteins contained WRKYGKK, WRKYDHK, or WRKYDQK domain. Seventeen genes were identified to group I, which contained two WRKY domains and had a zinc finger motif of C_2_H_2_ type (C-X_4_-C-X_22–23_-H-X_1_-H). Sixty-seven DcWRKY members contained a zinc finger motif of C_2_H_2_ type (C-X_4–5_-C-X_23_-H-X_1_-H), which were classified as group II. Group III had 11 members, which contained a C_2_HC(C-X_7_-C-X_23_-H-X_1_-C) zinc finger.

### Phylogenetic relationship and structure of the carrot DcWRKYs

**Figure 1:**
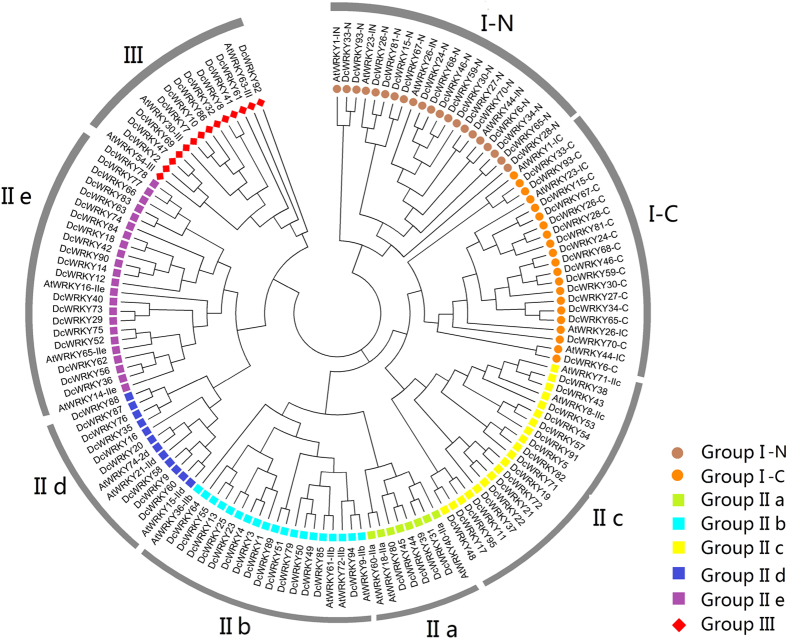
Phylogenetic tree of all WRKY proteins from carrot and *Arabidopsis.* Group I proteins with the suffix ‘N’ or ‘C’ indicates the N-terminal WRKY domains or the C-terminal WRKY domains.

To investigate the classification and phylogenetic relationship of the WRKY proteins in carrot and *Arabidopsis*, we used the domain region of the WRKY proteins from carrot and *Arabidopsis* to construct a phylogenetic tree. Based on the phylogenetic tree (Fig. 1), all the DcWRKY factors could be divided into three groups. Group I is an independent branch, whereas groups II and III showed a relatively close relationship. Group II, in particular, could be further divided into three main groups with five subgroups. Subgroups IIa and IIb were separated from one clade, and IIe and IId clustered to a branch.

**Figure 2:**
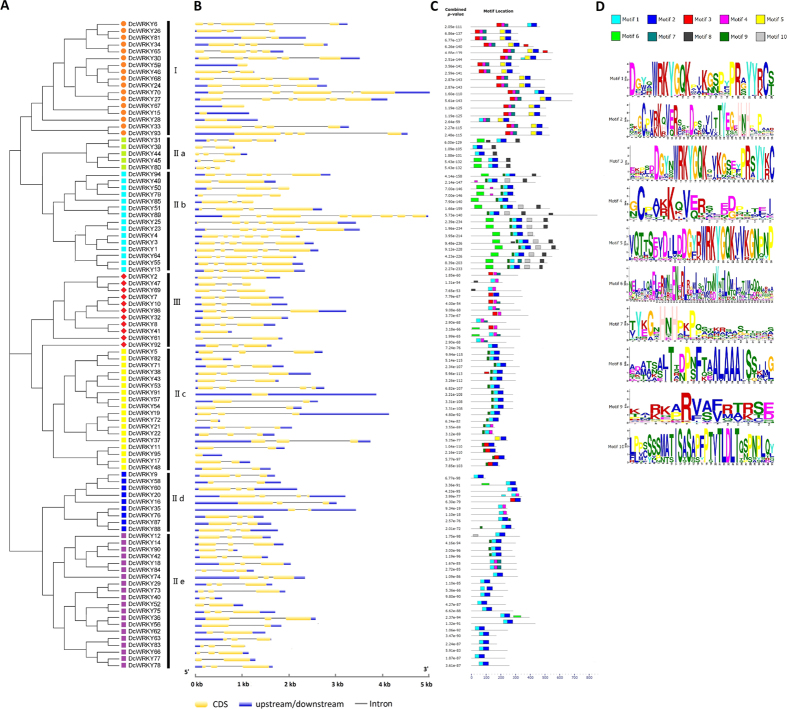
Structure analysis of carrot WRKY transcription factors. (**A**) NJ phylogenetic tree of DcWRKYs. (**B**) Exon–intron composition of *DcWRKY* genes. (**C**) Distribution of conserved motifs of DcWRKYs. (**D**) Logo of each motif. Different motifs are shown by different colors numbered 1 to 10. See legend for detailed color annotation.

The exon–intron structure analysis was performed to gain more insight into the *DcWRKY* genes. High variation was observed in numbers of exons and introns among *DcWRKY* genes (Fig. 2A,B). Forty-two *DcWRKYs* had two introns and accounted for the largest proportion, followed by eighteen *DcWRKYs* possessed only one intron and twelve *DcWRKYs* had three introns. *DcWRKY* genes belonging to the same group seemed to have similar exon–intron structures. For example, eight genes (*DcWRKY59*, *67*, *15*, *28*, *41*, *82*, *91*, *95*) did not contain any intron, and four of them (*DcWRKY59*, *67*, *15*, and *28*) were classified into group I, three of them (*DcWRKY82*, *91*, and *95*) were classified into group IIc. Group III contained eleven members, nine genes had two introns. For subgroups IIb, most genes contain four introns, while most IId and IIe genes had two introns. These results showed a strong correlation between exon–intron structure and phylogenetic relationship, which providing an additional foundation to support the classification.

The conserved motifs were predicted by MEME program to explore the diversity in each group. As illustrated in Fig. 2C,D, motifs 1, 3, and 5 contained a WRKYGQK sequence. The DcWRKY proteins that share a similar motif composition were clustered into the same group. For example, most members of groups IIa and IIb contained motifs 1, 2, 6, and 8, whereas group IId and IIe shared motifs 1 and 2. Motifs 1 and 2 were also present in group IIc. Motifs 2, 3, 4, 5, and 7 were present in group I, which contained two WRKY domains, whereas group III possessed motifs 3 and 4.

### Physicochemical analysis of deduced DcWRKY proteins

To analyze the physical and chemical characterizations of the carrot DcWRKYs, we calculated all 95 DcWRKY proteins using the Protparam tool (Table S1). The values of theoretical *pI* ranged from 4.61 to 10.04, and the average for all proteins was 7.24. No distinct difference was found between the percentage of positive and negative amino acids in all groups except for group IId, in which the percentage of positive amino acids was two-fold higher compared with negative amino acids. The content of aliphatic amino acids in all the proteins was very high, which accounted for an average of 16%, whereas the percentage of aromatic amino acids was only 7%. The average value of the aliphatic index reached 59.57, which suggested that the DcWRKY proteins contained rich aliphatic amino acids. Almost all DcWRKY proteins were calculated to be unstable proteins, only seven DcWRKY proteins were considered to be stable with aliphatic index values of less than 40. The grand average of hydropathicity of all DcWRKYs was less than zero, indicating that DcWRKY proteins were hydrophilic. Most of the physical/chemical properties of DcWRKY proteins were quite similar, but several differences were still observed, which may be due to the nonconserved regions in the protein sequences.

**Figure 3:**
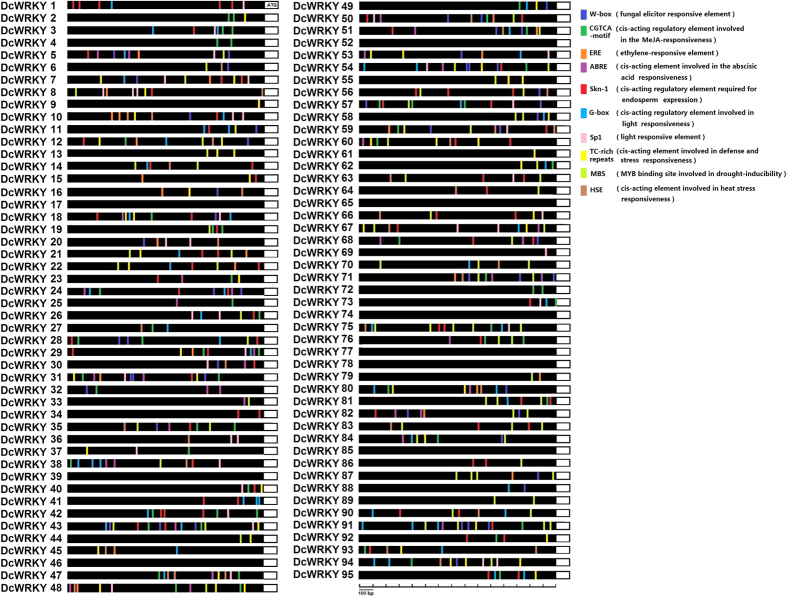
*cis* elements analysis of the promoter regions of carrot WRKY genes. The micro-segments in different colors were the putative elements sequence. The description of the ten *cis* elements were in brackets.

The *cis*-regulatory elements in all *DcWRKY* genes promoters were analyzed using the online software PlantCARE based the carrot genome data. A number of different kinds of *cis* elements were found, and the 10 most common elements were represented in Fig. 3. These elements included a fungal elicitor responsive element W-box, two light responsive elements (G-box and Sp1 elements), three hormone responsive elements (CGTCA-motif, ERF, and ABRE elements), a motif named Skn-1 associated with endosperm expression, a stress induction responding site TC-rich, a drought responsive element MBS, and a heat stress responsive site HSE. Most *DcWRKY* genes contained more than one *cis* element in their promoter regions. WRKY proteins usually functioned as transcriptional regulators by binding to W-box ((C/T)TGAC(T/C)) to regulate defense-related genes^11^. We found that several *DcWRKY* genes also contained W-box element in their promoter regions. The same findings were identified in Chinese cabbage and *Arabidopsis*^31^,^32^, suggesting that these *DcWRKY* genes may be regulated by other *DcWRKY* genes or self-regulated mechanisms. A lot of studies have also reported that WRKY factors were responsive to various stresses including drought, cold and salinity^14^,^33^, that may due to the upstream genes specificity bind the corresponding *cis* element to regulate the expression of *WRKY* genes.

### Subcellular localization of DcWRKYs

**Figure 4:**
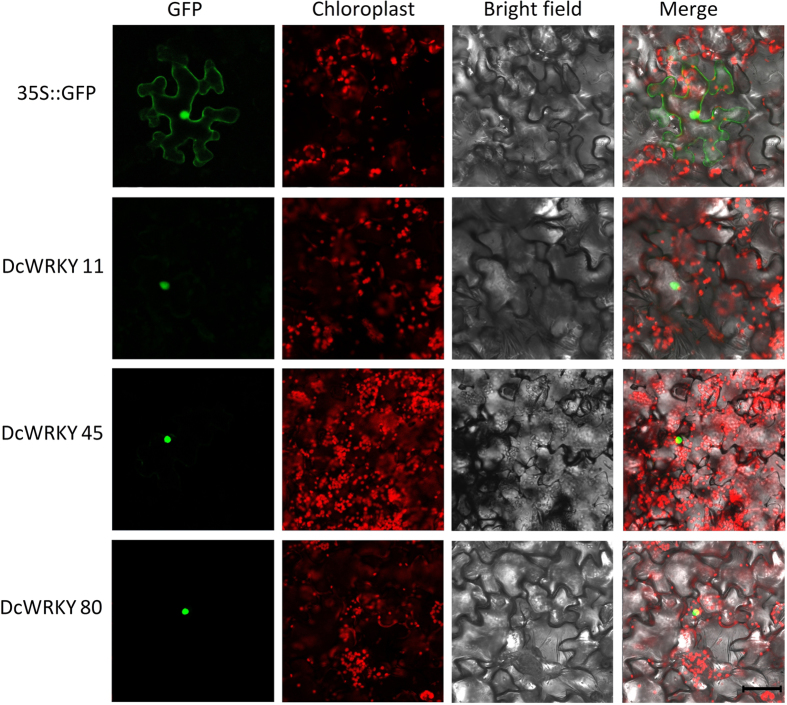
Subcellular localization of carrot WRKYs. The DcWRKY-GFP fusion plasmids were transiently expressed in tobacco cells. GFP fuorescence was localized in the nucleus. Bar = 50 μM.

Subcellular location analysis on the combination of WoLF PROST and TargetP showed that most DcWRKY proteins were located in the nucleus (Table S1). To examine the subcellular localization of DcWRKY proteins, the coding sequences of three predicted nucleus-localized WRKYs were fused to the N-terminus of GFP and expression in tobacco cells *via* biolistic bombardment. The GFP fluorescence was observed only in the nucleus of transformant cells (Fig. 4), indicating that DcWRKY45, DcWRKY11 and DcWRKY80 were localized to the nucleus *in vivo*.

### Evolution and distribution of WRKY family factors among different species

**Figure 5:**
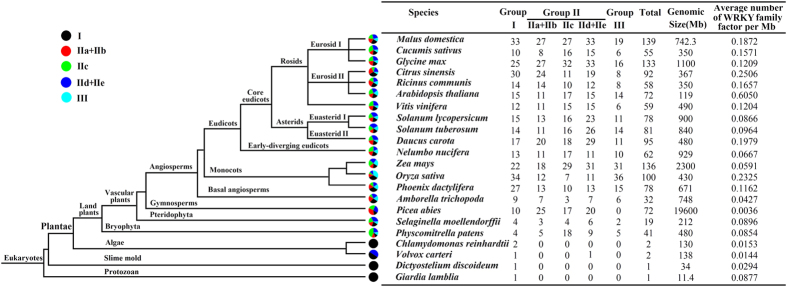
Schematic of species phylogenetic relationships. The distributions of WRKY transcription factors among different species are compared. Each color represents a WRKY subgroup and the colored section represents the proportion of this subgroup.

The WRKY family factors are commonly found in plants, but are also reported in two nonphotosynthetic organisms, namely, *D. discoideum*^14^ and *G. lamblia*^34^. We constructed a schematic of the phylogenetic tree in eukaryote evolution. Based on the whole-genome level, the number of WRKYs in each species was counted (Fig. 5). In *D. discoideum* and *G. lamblia*, only one WRKY protein was identified in both organisms. Similarly, only two WRKY proteins were identified in the algae *Chlamydomonas reinhardtii* and *Volvox carteri*. However, land plants have a relatively large number of WRKY family factors. In addition, species that have a larger genome seem to contain a greater number of WRKY transcription factors except for *P. abies*. In all species, the densities of WRKY proteins in *A. thaliana* (0.6050 number/Mb) were the highest, followed by *Citrus sinensis* (0.2508 number/Mb), *Oryza sativa* (0.2325 number/Mb), and *D. carota* (0.1979 number/Mb), which were higher than those in lower plants. The results of evolutionary analysis imply that a sharp expansion occurred in the evolutionary process from low plants to high plants, and the density of WRKY proteins increased as the plants evolved. Notably, group I existed in all species, whether in plantae, slime mold, or protozoans, suggesting that this group evolved early and represents the ancestral form. The number of group III in *P. abies*, *S. moellendorffii*, and *P. patens* was less than the other higher plants. However, the number of this group was higher in monocots than in eudicots.

### Identification of orthologous and paralogous *WRKY* genes in plants

To survey the orthologous and paralogous *WRKY* genes among different species, we performed a comparative study of nine divergent taxa in lower and higher plants. The genome sequences of alga (*C. reinhardtii*), moss (*P. patens*), fern (*S. moellendorffii*), *P. abies*, *Arabidopsis*, carrot, grapevine, apple, and rice have been completed. A total of 73 homologous gene groups were obtained by OrthoMCL software, containing 543 paralogous, 452 orthologous, and 677 coorthologous gene pairs. Results of the comparison between different species are presented in Table 2. A large number of paralogous gene pairs were found in *P. abies* (237), followed by apple (102), *P. patens* (67), and carrot (56). Species with large genome seems to show correspondingly great paralogous genes, except for grape. Grape had only seven paralogous gene pairs. A total of 28/23 and 21/19 orthologous/coorthologous gene pairs were identified in carrot–rice and carrot–*Arabidopsis*. Numerous orthologous/coorthologous gene pairs were also found between carrot and other dicotyledons. However, a small number of homologous genes were found between the gymnosperm *P. abies* and other plants, although the former has a relatively large WRKY family (72 WRKY transcription factors) and has the most number of paralogous gene pairs (237 pairs).

### Interaction network of DcWRKY proteins in carrot

**Figure 6:**
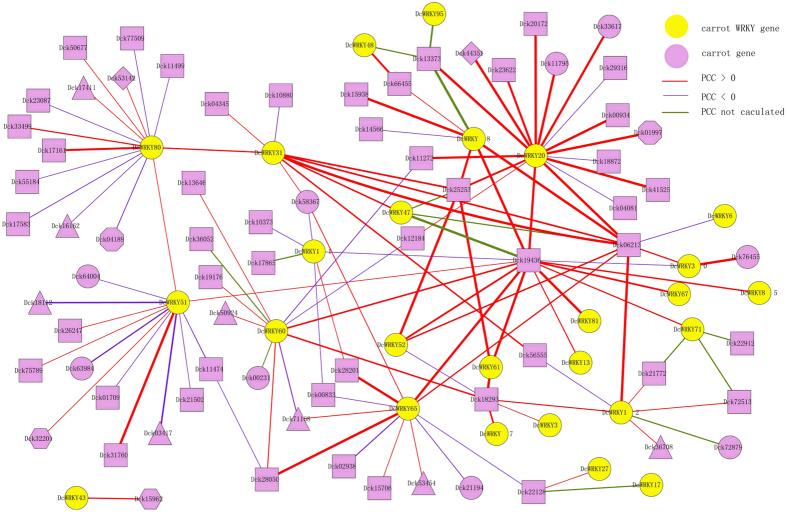
Interaction network of WRKY factors in carrot according to the orthologs in *Arabidopsis*. PCC: Pearson correlation coefficient. Different graphical represent the subcelluar location of different proteins. Ellipse: Nucleus; Rectangle: Unclear; Triangle: Plastid; Diamond: Vacuole; Hexagon: Unclear.

Pearson correlation coefficients (PCC value) were calculated among carrot proteins based on the orthologs in *Arabidopsis* to verify the co-expression relationships between DcWRKY factors and other carrot proteins. A total of 38 DcWRKY proteins showed interactions with other proteins in carrot genome and 124 orthologs pairs were identified (Fig. 6). Among them, 75 orthologs pairs showed positive correlations (PCC value > 0) and 35 orthologs pairs showed negative correlations (PCC value < 0). In addition, the PCC values of 14 proteins could not be calculated. Three pairs of DcWRKY proteins, namely, DcWRKY80 (group IIa)/DcWRKY31 (group IIa), DcWRKY81 (group IIa)/DcWRKY50 (group IIb), and DcWRKY31 (group IIa)/DcWRKY47 (group III), showed co-expression relationships with positive correlations. These relationships indicate that the proteins that belonged to the same group may have the same functions in the regulation network. DcWRKY20 protein, which belonged to group IId, showed significant correlations with 17 proteins, indicating that DcWRKY20 probably plays an important role in biological regulation mechanisms by inducing other genes. Moreover, two DcMAPK proteins Dck19436 (DcMAPK1) and Dck06213 (DcMAPK4) respectively showed potential regulation relationships with nine DcWRKY proteins, including DcWRKY20.

**Figure 7:**
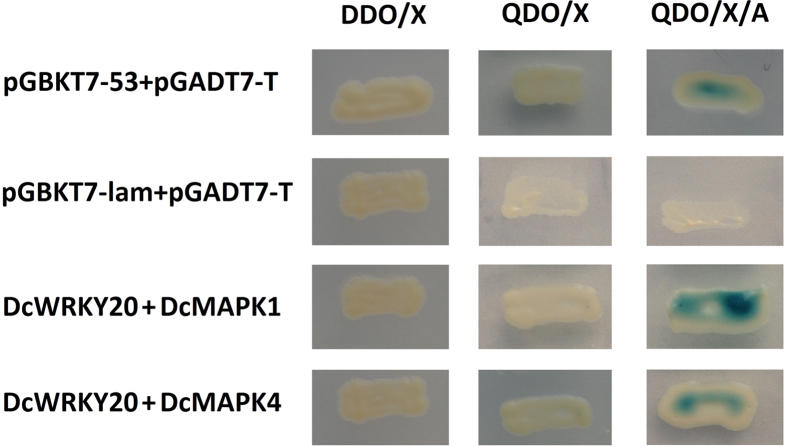
Interaction between DcWRKY20 and DcMAPK1/DcMAPK4 in yeast cells. Transformants grown on DDO, QDO, and QDO/X/A. DDO: SD/-Leu/-Trp; QDO: SD/-Ade/-His/-Leu/-Trp; QDO/X/A: QDO with X-a-gal and aureobasidin A.

To assess whether there were interactions between DcWRKY20 and two DcMAPK proteins, the fusion plasmids in the vectors pGADT7 and pGBKT7 were co-transformed into the yeast strain AH109 and the interaction was quantified with X-α-Gal assays. Figure 7 showed that co-expression of the DcWRKY20 with DcMAPK1 or DcMAPK4 resulted in X-α-galactosidase expression activity. The positive control showed that pGBKT7-53 can interact with pGADT7-T, and the negative control did not display such expression. These results demonstrated that DcWRKY20 interacted directly with DcMAPK1 and DcMAPK4 in yeast.

### Expression analysis of *DcWRKY* genes during root development in carrot

**Figure 8:**
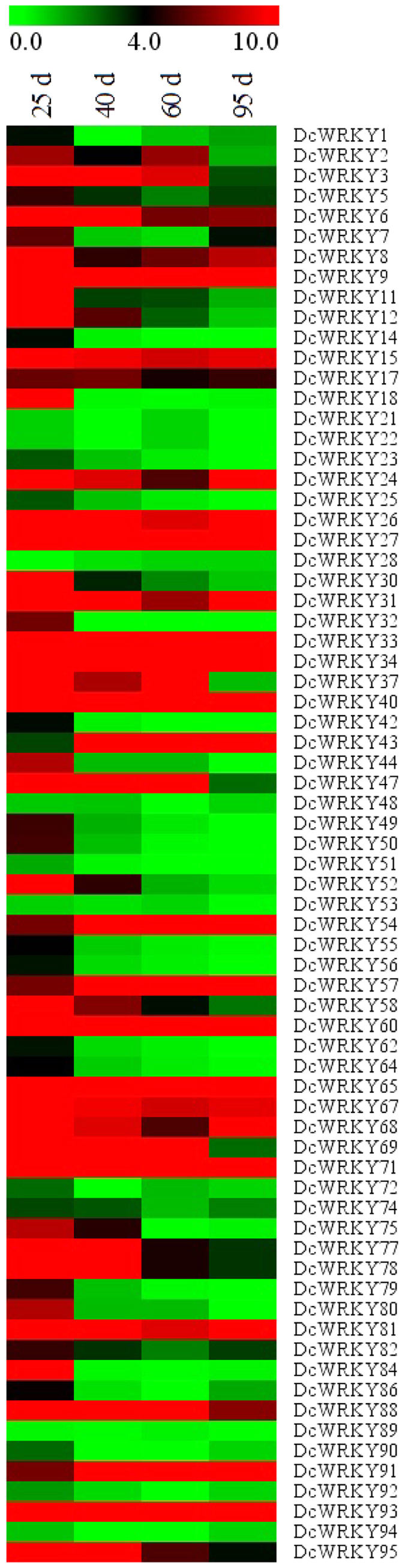
Transcript abundances of *DcWRKY* genes at different developmental stages (25 d, 40 d, 60 d, and 95 d) in carrot root.

The transcript abundances of *DcWRKY* genes during development in carrot were analyzed through the calculation of transcriptome data (SRR2177455). All *DcWRKY* genes were surveyed and 71 *DcWRKY* genes were found to have the expression. As showed in Fig. 8, the *DcWRKY* genes showed a broad expression during different developmental stages. Some genes, like *DcWRKY6*, *8*, *24*, *31*, *88*, and *95*, showed relatively high expression levels in all stages, while *DcWRKY1*, *23*, *53*, and *89* expressed very low. Throughout the Fig. 7, we found that the stage with 25 d had a large difference compared to other three stages, while a similar pattern was observed between 60 d and 90 d.

**Figure 9:**
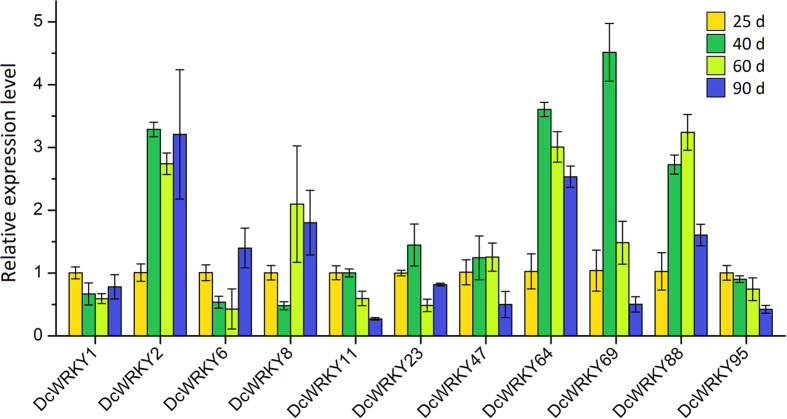
Expression profiles of *DcWRKY* genes at different developmental stages (25 d, 40 d, 60 d, and 95 d) in carrot root.

The WRKY genes in *Arabidopsis*, such as *AtWRKY6*^35^, *AtWRKY44*^36^, and *AtWRKY75*^37^, which have been confirmed to play important roles in plant development. According to our homologous analysis and transcriptome data, several *DcWRKY* genes were selected for qRT-PCR validation. As illustrated in Fig. 9, the expression patterns of those eleven *DcWRKY* genes had a greater difference at four development stages. Meanwhile, the qRT-PCR analysis and transcriptome data of most genes were consistent. The expression levels of four genes (*DcWRKY2*, *64*, *69*, and *88*) increased over the process of carrot development. Among them, *DcWRKY2* and *DcWRKY64* showed significant expression changes at the initial stage and maintained higher expression levels at the later stages, while the expression of two others (*DcWRKY69* and *DcWRKY88*) was highly variable. *DcWRKY11* and *DcWRKY95* exhibited a decrease pattern of expression at all four stages (Fig. 8), and qRT-PCR verified this result (Fig. 9). The expression levels of *DcWRKY6* and *DcWRKY8* at the initial stage were significantly decreased, while they showed a significant increase in the later stages.

### Expression profiles of *DcWRKY* genes under biotic stresses in carrot

**Figure 10:**
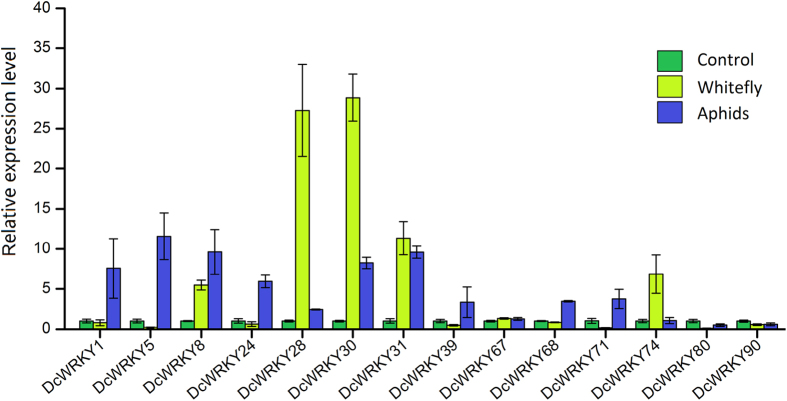
Expression profiles of *DcWRKY* genes under biotic stresses (whitefly and aphid tests) in carrot.

In regards to *DcWRKY* genes response to biotic stresses, fourteen *DcWRKY* genes which their orthologous genes in *Arabidopsis* involved in biotic stress were selected to determine the expression profiles after treatment with whitefly and aphids infections, respectively. As shown in Fig. 10, those selected *DcWRKY* genes were sensitive to biotic stresses. Nine of the tested *DcWRKY* genes exhibited a high level of accumulation after subjected to aphid stress, especially *DcWRKY5*, followed by *DcWRKY31*, *8*, *30* and *1*. The expressions of *DcWRKY8*, *28*, *30*, *31* and *74* were induced after inoculation with whitefly. Among them, the effect whitefly infection on *DcWRKY28* and *DcWRKY30* response were much stronger than others. *DcWRKY8*, *30*, and *31* were found to be both significantly upregulated in two biotic stresses, whereas weak expressions were observed for *DcWRKY80* and *DcWRKY90*.

### Expression profiles of *DcWRKY* genes under abiotic stresses in carrot

**Figure 11:**
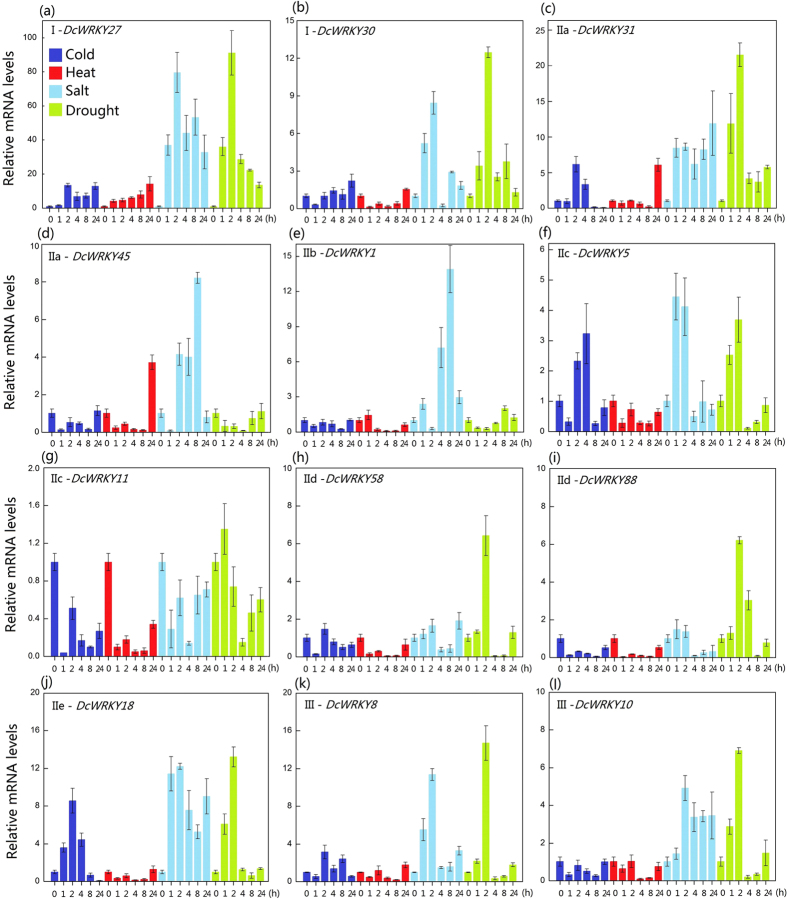
Expression patterns of *DcWRKY* genes under heat, cold, salt, and drought stresses. Samples collected at 0, 1, 2, 4, 8 and 24 h after each treatment.

Previous studies reported that *WRKY* genes are involved in abiotic stress resistance^22^,^38^. Basing on the transcriptome data in *Arabidopsis* under different abiotic stresses^39^ and the orthologous genes between *DcWRKYs* and *AtWRKYs* (Table 1), we selected 12 *DcWRKY* genes from each group to analyze their expression patterns under four abiotic stresses (cold, heat, salt, and drought) in carrot. Figure 11 shows that the members of group I, *DcWRKY27* and *DcWRKY30*, had only one motif difference in structure but exhibited different responses to abiotic stresses.

In heat and cold treatments, *DcWRKY27* was evidently upregulated and maintained a high expression level during 24 h, whereas *DcWRKY30* was not sensitive to temperature treatment. Moreover, the expression level of *DcWRKY27* also increased in salt and drought treatments; the expression level in salt treatment increased by almost 80 times. However, the trend in the expression of *DcWRKY30* initially increased and reached a maximum after 2 h and then decreased in salt and drought conditions. Based on the further classification of group II (IIa+IIb, IIc, and IId+IIe), we found that the phenomenon of the same subgroup genes that showed a similar expression trend was common in group II, for example, *DcWRKY45* (IIa)/*DcWRKY1* (IIb), *DcWRKY5* (IIc)/*DcWRKY11* (IIc), and *DcWRKY58* (IId)/*DcWRKY88* (IId). The members of group II seemed to be more sensitive to drought and salt stresses. In particular, under salt stress, *DcWRKY1* and *DcWRKY18* increased by 14- and 12-fold, respectively.

*DcWRKY8* and *DcWRKY10*, which both belonged to group III, had a motif difference. The expression levels of the *DcWRKY8* and *DcWRKY10* genes slightly changed under cold and heat conditions. Moreover, *DcWRKY8* responded rapidly to salt stress response, but both genes had a similar change trend under drought treatment: an initial increase and peak at 2 h followed by a decrease.

## Discussion

Increasing studies have confirmed that WRKY transcription factors are involved in plant growth and development, signal transduction, and stress response. Studies on functions of WRKY factors were conducted on model plants, such as *Arabidopsis*^15^ and rice^9^, but almost no reports on carrot, an Apiaceae plant, have been found. In this study, 95 genes were identified to encode WRKY transcription factors, and they were divided into three groups based on the similarity of structure and motif. Among all motifs, motifs 1, 3, and 5 contained a WRKYGQK sequence. Motifs 3 and 5 were present in group I members, while motif 1 was found in groups II and III. In addition, comparative structural analysis of DcWRKYs revealed that DcWRKYs in the same group shared similar exon–intron structures. The analysis on structures of *DcWRKY* genes might provide a way to find out which group of *WRKY* genes might be of a more ancient origin.

A phylogenetic tree of WRKY transcription factors from carrot and the dicotyledonous model plant *Arabidopsis* was constructed. The result was consistent with domain and zinc finger type classifications of carrot WRKY transcription factors. Basing on the current genomic data, we built a model diagram for the origin and evolution of WRKY family transcription factors. Some species that can represent different branches on the plant’s evolutionary tree were selected to analyze the WRKY family. As illustrated in Fig. 5, group I existed in all species, including the two nonplant species *G. lamblia* and *D. discoideum*. However, groups II and III only seemed to be specific for the green plant lineage and expand with the evolution of higher plants^17^,^40^. The results expound and support that all the WRKY groups may have evolved prior to the moss lineage. Group I may have originated before the origin of eukaryotes about 1.5 billion years ago^14^.

The events of gene duplication and loss are the driving forces during species evolution. With genome amplification, the number and density of WRKY factors are greatly increased. The algae *C. reinhardtii* and *V. carteri* both contain only two WRKY factors, whereas the moss *P. patens* and the fern *S. moellendorffii* have 41 and 19 WRKY members, respectively. Groups II and III appeared before the origin of the moss plant and have been duplicated many times with plant evolution, which accounted for a large proportion in higher plants. In this study, we selected several monocotyledonous and dicotyledonous plants to compare the duplication and evolution of WRKY factors. The duplication of WRKY factors in monocotyledonous and dicotyledonous plants was independent, and the duplication and diversification of WRKY factors occurred before the differentiation of monocots and eudicots. The number of group III members in monocotyledonous plants was evidently higher than that in dicotyledonous plants. This result indicates that group III seems to be more active in duplication and may have more function in monocots. Gene replication has been confirmed as one of the reasons that lead to new gene functions^41^. Therefore, we can infer that WRKY factors were expended during plant evolution. The unique gene duplication events in different species revealed the species specificity in the evolution of WRKY factors.

The homologous *WRKY* genes were compared to further analyze the genetic relationship. A total of 73 homologous gene groups belonging to nine distinctly divergent groups were identified based on the sequence similarity. The number of homologous genes showed a significant difference among different species, which may be influenced by the genetic distance between the analyzed species. Numerous homologous gene pairs were found between carrot and other dicotyledons because of their close relationship. However, few homologous gene pairs were identified between carrot and other monocotyledonous and lower plants. This study also shows that species with large genome indicates more paralogous genes. This phenomenon may be contributed by the plant’s whole genome duplication (WGD). The WGD event is an important evolutionary feature of plant genome, which can explain the results of gene duplication or loss^42^,^43^. Previous studies showed that *Arabidopsis* has undergone three WGD, and rice also has undergone at least one WGD event^5^,^44^. Furthermore, the number of paralogous genes in grapevine is much less than that in other higher species, which could be due to grapevines’ failure to undergo any WGD event^45^. Carrot may also have experienced one or more rounds of WGD events, which could have led to the DcWRKY factors being expended.

Previous studies founded that WRKY transcription factors have complex regulatory networks in response to biotic and abiotic stresses. Among the networks, the WRKY factors are controlled by different levels, including direct positive or negative control by WRKY factors, regulation *via* other transcription factors or proteins, and the small-RNA-WRKY interactome^21^. In this study, an interaction network between DcWRKYs and other carrot proteins was constructed. Thirty-eight DcWRKY proteins showed co-expression relationships with other proteins in carrot genome, indicating that these DcWRKYs interact with other proteins to modulate stress resistance. The DcWRKY20, was identified in a yeast-two-hybrid system that interacted directly with two mitogen-activated protein kinases DcMAPK1 and DcMAPK4. In *Arabidopsis*, MAPKs are important regulating factors and act by phosphorylating transcription factors, which subsequently activates transcription of other genes, including WRKY genes^46^,^47^. In *Arabidopsis*, WRKY33 was subject to post-translational modification by MAPK4 that was involved in pathogens and SA-mediated responses^48^. The phosphorylation of OsWRKY30 by MAPKs was crucial in order for OsWRKY30 to perform its biological function in rice^49^. Here we found that DcWRKY20 can interact with MAPKs, speculating that DcWRKY20 activity may be regulated by post-translational modifications with MAPKs. We also confirmed the W-box binding activity of several DcWRKY factors by yeast one-hybrid system. However, no obviously interactions of these DcWRKYs and the W-box element were observed in yeast cells (data not shown), suggesting that they may not have direct control relationship. Also the absence of interaction with W-box in yeast may be due to the absence of required co-factors, such as phosphorylation^11^, zinc-ions^50^.

Evidence has demonstrated that WRKY transcription factor are involved in plant growth and development. The WRKY transcription factor in rice, *OsWRKY78*, can regulates stem elongation and seed development^51^. Overexpression *OsWRKY31* gene enhances disease resistance and affects root growth and auxin response in transgenic rice plants^52^. *GhWRKY15*, a member of the WRKY family identified from cotton, is involved in disease resistance and plant development^53^. Expression analyses in carrot root development helped to screen *WRKY* genes which may be involved in carrot root development. The *DcWRKY3*, *DcWRKY8 DcWRKY24*, *DcWRKY64*, and *DcWRKY88*, were abundantly expressed at all four stages of development in carrot root. Notably, the orthologous genes of *DcWRKY3* and *DcWRKY8* in *Arabidopsis*, *AtWRKY6* (AT1G62300.1) and *AtWRKY53* (AT4G23810.1), which have been confirmed to play an important role in leaf development^35^,^54^. This result suggested that *DcWRKY3* and *DcWRKY8* may have similar functions to their orthologous *Arabidopsis* genes during plant development.

Transcriptome analysis revealed that a large number of drought, cold, and high-salinity stress-responsive genes, including numerous *WRKY* genes, were identified in *Arabidopsis*^39^. The comparative analysis of *DcWRKY* genes with their homologous *AtWRKY* genes helped to predict the potential functions of DcWRKY proteins. Following homologous gene annotations in *Arabidopsis*, we deduced the functional roles of *DcWRKY*s. A qRT-PCR experiment showed that the expression profile of *DcWRKY* genes agreed well with the *WRKY* genes in *Arabidopsis*. The two *Arabidopsis WRKY* genes *AtWRKY33* (AT2G38470.1) and *AtWRKY40* (AT1G80840.1) were induced by biotic and abiotic stresses^55^,^56^. Their orthologous genes in carrot are *DcWRKY30* and *DcWRKY31*, which were also found to be evidently upregulated under drought, salt and pathogenic stresses. As plants evolved from lower to higher, plants have established a series of mechanisms of plant growth and development, metabolic regulation, and stress response. The rapid expansion of the *WRKY* gene family may be a way to meet the requirements for these pathways. Most stress-resistance traits are often controlled by multiple genes^57^,^58^. The expression patterns of several *DcWRKY* genes significantly changed, indicating that these genes all appeared to be involved in biotic and abiotic stress response. We expect that future research will reveal the accurate regulation mechanisms of *WRKY* genes in signaling pathways and stress responses.

## Materials and Methods

### Identification of putative *WRKY* genes in carrot

The genome sequences of carrot (*Daucus carota* L. cv. Kuroda) were downloaded from the Carrot Genome Project web site (http://apiaceae.njau.edu.cn/carrthe otdb/index.php)^30^, and the transcriptome sequences were downloaded from NCBI SRA database^59^ and Carrot Genome Project web site^30^. The hidden Markov model (HMM) of the WRKY domain (PF03106) was downloaded from the Sanger database (http://pfam.sanger.ac.uk/family/WRKY). All the putative carrot DcWRKY factors were obtained *via* screening carrot genome sequences and transcriptome sequences by HMMER 3.0 software (http://hmmer.janelia.org/) using default parameters. The sequences were then submitted to the NCBI database (http://ncbi.nlm.nih.gov/) to search for WRKY domain. All the nonredundant gene sequences encoding complete WRKY domains were considered as putative *WRKY* genes.

### Sequence alignments and phylogenetic analysis

The database of the *Arabidopsis* WRKY family factors was downloaded from the plant transcription factor database (http://www.arabidopsis.org/). The WRKY family databases of other species were downloaded from the plant transcription factor database (http://planttfdb.cbi.pku.edu.cn/)^3^. All WRKY factors were classified into subgroups based on the sequence alignments of WRKY proteins in carrot and *Arabidopsis* using ClustalW with default parameters^60^. The domain region of the WRKY proteins from carrot and Arabidopsis were used to construct a phylogenetic tree with MEGA5.0 using the neighbor-joining method with 1000 bootstrap replicates^61^.

### Characterization of conserved motif distributions and structures of DcWRKY factors

Analysis of the exon-intron organization of *DcWRKY* genes was performed by comparing coding sequences with their corresponding genomic sequences using the GSDS software (http://gsds.cbi.pku.edu.cn)^62^. Conserved motifs for each deduced DcWRKY amino acid sequence were analyzed by MEME Suite (version4.9.0; http://meme.nbcr.net/meme/)^63^. The parameters were set as follows: maximum number, 20; minimum width, 10; and maximum width, 50. The compositions as well as physical and chemical characterizations of deduced DcWRKY proteins were analyzed by the Protparam tool (http://web.expasy.org/protparam) and the Sequence Manipulation Suite (http://www.bio-soft.net/sms/). The subcellular locations were predicted using WoLF PSORT (http://wolfpsort.org)^64^ and TargetP1.1 server (http://www.cbs.dtu.dk/services/TargetP)^65^. The upstream 1,500 bp regions of all *DcWRKY* genes were analyzed to determine the *cis*-regulatory elements by using the plant database PlantCARE (http://bioinformatics.psb.ugent.be/webtools/plantcare/html/)^66^.

### Subcellular localization

To test the predicted nuclear localization patterns, the full length of three *DcWRKY* genes (*DcWRKY11*, *DcWRKY45*, *DcWRKY80*) without the stop codon were amplified with primers as shown in Table S2. The PCR products were inserted into a GFP-fusion expression vector PA7, and transferred to tobacco leaves to determine the subcellular localization. Empty vector *35S::GFP* was used as control. Fluorescence images of GFP fusion proteins were observed using the LSM780 confocal microscopy imaging system (Zeiss, Germany).

### Identification of orthologous and paralogous WRKY factors

The orthologous, paralogous, and coorthologous WRKY factors in alga (*C. reinhardtii*), moss (*P. patens*), fern (*S. moellendorffii*), *Picea abies*, *Arabidopsis*, carrot, grape, apple, and rice were identified using OrthoMCL (http://orthomcl.org/orthomcl/) with default settings^67^. The interaction network associated with the *Arabidopsis* WRKY orthologous factors in carrot was constructed using the *Arabidopsis* Interactions Viewer and cytoscape software^68^.

### Yeast two-hybrid assay

For the yeast two-hybrid assay, the DcWRKY20 cDNA fragment was cloned into the pGBKT7 vector, and the DcMAPK1 and DcMAPK4 cDNA fragments were cloned into the pGADT7 vector, respectively. Unique amplified primers for cloning these three genes were represented in Table S2. These vectors were co-transformed into the yeast AH109 strain, and the transformants were identified with X-α-gal according to a manufacture’s protocol (Clontech, Takara, Dalian, China). Yeast co-transformation with pGBK7-53 and pGADT7-T was used as a positive interaction control, and co-transformation with pGBKT7-Lam and pGADT7-T was used as a negative control.

### Transcript abundance analysis

Transcript abundance analysis in carrot root development was based on transcriptome data (SRR2177455). Transcript abundance analysis of *DcWRKY* genes were estimated by calculating read density as ‘reads per kilobase of exon model per million mapped reads’ (RPKM)^69^. The heatmap was performed by Multiexperiment Viewer software (http://www.tm4.org/)^70^.

### Plant materials, abiotic and biotic treatments, and qRT-PCR

Experimental samples were obtained from two-month-old ‘Kurodagosun’ carrot seedlings, which were grown in pots in a controlled-environment growth chamber. The temperature was set at 4 °C for the cold treatment and 38 °C for the heat treatment. Salt and drought treatments were carried out by irrigating with 200 mM NaCl and 20% PEG6000, respectively. Seedlings irrigated with sterile water were used as blank control. For plant biotic damage, whitefly was mechanically inoculated on the carrot leaves by rubbing the leaf with the virus, and adult aphids settled on carrot plants for the aphid tests. After 7 days, infective-stage leaves were collected and used for bioassays. All samples were immediately frozen in liquid nitrogen and stored at −70 °C.

Total RNAs were extracted from the samples using the total RNA kit (Tiangen, Beijing, China) and then reverse transcribed into cDNAs using the PrimeScript RT reagent kit (TaKaRa, Dalian, China). Three independent PCR reactions were carried out for each gene using the MyiQ Single-Color RT-PCR detection system (Bio-Rad, Hercules, CA, USA). The PCR amplification profile was as follows: 95 °C for 30 s and 40 cycles of 94 °C for 5 s and 60 °C for 30 s, at last a melting curve (65–95 °C, at increments of 0.5 °C) was generated to check the amplification specificity. The relative gene expression was calculated with the 2^−ΔΔCT^ method^71^. All primers used are shown in Table S2. The *DcTUB* gene was used as an internal control to normalize the expression of *DcWRKY* genes.

## Additional Information

**How to cite this article**: Li, M.-Y. *et al.* Genomic identification of WRKY transcription factors in carrot (*Daucus carota*) and analysis of evolution and homologous groups for plants. *Sci. Rep.* **6**, 23101; doi: 10.1038/srep23101 (2016).

## Supplementary Material

Supplementary Information

## Figures and Tables

**Table 1:** Identified *DcWRKY* genes and their related information.

Gene name	ORF (aa)	Group	Conserved motif	Domain pattern	Best hit to *Arabidopsis*		AtWRKYs related to abiotic stresses
					Gene name	Locus ID	
DcWRKY1	598	IIb	WRKYGQK	C-X_5_-C-X_23_-HXH	AtWRKY6	AT1G62300.1	cold, drought, salt
DcWRKY2	318	III	WRKYGQK	C-X_7_-C-X_23_-HTC	AtWRKY70	AT3G56400.1	
DcWRKY3	566	IIb	WRKYGQK	C-X_5_-C-X_23_-HXH	AtWRKY6	AT1G62300.1	cold, drought, salt
DcWRKY4	436	IIb	WRKYGQK	C-X_5_-C-X_23_-HXH	AtWRKY6	AT1G62300.1	cold, drought, salt
DcWRKY5	287	IIc	WRKYGQK	C-X_4_-C-X_23_-HXH	AtWRKY48	AT5G49520.1	drought, salt
DcWRKY6	472	I	WRKYGQK	C-X_4_-C-X_23_-HXH	AtWRKY44	AT2G37260.2	
DcWRKY7	351	III	WRKYGQK	C-X_7_-C-X_23_-HXC	AtWRKY41	AT4G11070.1	
DcWRKY8	332	III	WRKYGQK	C-X_7_-C-X_23_-HXC	AtWRKY53	AT4G23810.1	drought
DcWRKY9	101	IId	WRKYGQK	C-X_5_-C-X_23_-HXH	AtWRKY15	AT2G23320.1	drought, salt
DcWRKY10	294	III	WRKYGQK	C-X_7_-C-X_22_-HXC	AtWRKY30	AT5G24110.1	drought, salt
DcWRKY11	175	IIc	WRKYGQK	C-X_4_-C-X_23_-HXH	AtWRKY75	AT5G13080.1	salt
DcWRKY12	332	IIe	WRKYGQK	C-X_5_-C-X_22_-HXH	AtWRKY22	AT4G01250.1	drought, salt
DcWRKY13	520	IIb	WRKYGQK	C-X_5_-C-X_23_-HXH	AtWRKY6	AT1G62300.1	cold, drought, salt
DcWRKY14	305	IIe	WRKYGQK	C-X_5_-C-X_22_-HXH	AtWRKY22	AT4G01250.1	drought, salt
DcWRKY15	514	I	WRKYGQK	C-X_4_-C-X_22_-HXH (N)/C-X_4_-C-X_23_-HXH (C)	AtWRKY4	AT1G13960.1	drought, salt
DcWRKY16	338	IId	WRKYGQK	C-X_5_-C-X_23_-HXH	AtWRKY21	AT2G30590.1	
DcWRKY17	242	IIc	WRKYGQK	C-X_4_-C-X_23_-HXH	AtWRKY24	AT5G41570.1	
DcWRKY18	311	IIe	WRKYGQK	C-X_5_-C-X_23_-HXH	AtWRKY22	AT4G01250.1	drought, salt
DcWRKY19	204	IIc	WRKYGKK	C-X_4_-C-X_23_-HXH	AtWRKY51	AT5G64810.1	cold
DcWRKY20	346	IId	WRKYGQK	C-X_5_-C-X_23_-HXH	AtWRKY21	AT2G30590.1	
DcWRKY21	163	IIc	WRKYGKK	C-X_4_-C-X_23_-HXH	AtWRKY50	AT5G26170.1	
DcWRKY22	157	IIc	WRKYGKK	C-X_4_-C-X_23_-HXH	AtWRKY50	AT5G26170.1	
DcWRKY23	522	IIb	WRKYGQK	C-X_5_-C-X_23_-HXH	AtWRKY6	AT1G62300.1	cold, drought, salt
DcWRKY24	507	I	WRKYGQK	C-X_4_-C-X_22_-HXH(N)/C-X_4_-C-X_23_-HXH (C)	AtWRKY33	AT2G38470.1	drought, salt
DcWRKY25	532	IIb	WRKYGQK	C-X_5_-C-X_23_-HXH	AtWRKY6	AT1G62300.1	cold, drought, salt
DcWRKY26	324	I	WRKYGQK	C-X_4_-C-X_22_-HXH(N)/C-X_4_-C-X_23_-HXH (C)	AtWRKY3	AT2G03340.1	
DcWRKY27	691	I	WRKYGQK	C-X_4_-C-X_22_-HXH(N)/C-X_4_-C-X_23_-HXH (C)	AtWRKY2	AT5G56270.1	heat
DcWRKY28	298	I	WRKYGQK	C-X_4_-C-X_22_-HXH (N)/C-X_4_-C-X_23_-HXH (C)	AtWRKY33	AT2G38470.1	drought, salt
DcWRKY29	233	IIe	WRKYGQK	C-X_5_-C-X_23_-HXH	AtWRKY20	AT1G29280.1	
DcWRKY30	551	I	WRKYGQK	C-X_4_-C-X_22_-HXH (N)/C-X_4_-C-X_23_-HXH (C)	AtWRKY33	AT2G38470.1	drought, salt
DcWRKY31	343	IIa	WRKYGQK	C-X_5_-C-X_23_-HXH	AtWRKY40	AT1G80840.1	drought, salt
DcWRKY32	390	III	WRKYGQK	C-X_7_-C-X_23_-HXC	AtWRKY30	AT5G24110.1	drought, salt
DcWRKY33	531	I	WRKYGQK	C-X_4_-C-X_22_-HXH (N)/C-X_4_-C-X_23_-HXH (C)	AtWRKY1	AT2G04880.2	
DcWRKY34	429	I	WRKYGQK	C-X_4_-C-X_22_-HXH (N)/C-X_4_-C-X_23_-HXH (C)	AtWRKY20	AT4G26640.1	
DcWRKY35	248	IId	WKKYDHK	C-X_5_-C-X_23_-HXH	AtWRKY7	AT4G24240.1	salt
DcWRKY36	397	IIe	WRKYGQK	C-X_5_-C-X_23_-HXH	AtWRKY14	AT1G30650.1	
DcWRKY37	250	IIc	WRKYGQK	C-X_4_-C-X_23_-HXH	AtWRKY13	AT4G39410.1	
DcWRKY38	305	IIc	WRKYGQK	C-X_4_-C-X_23_-HXH	AtWRKY71	AT1G29860.1	
DcWRKY39	179	IIa	WRKYGQK	C-X_5_-C-X_22_-HXH	AtWRKY40	AT1G80840.1	drought, salt
DcWRKY40	219	IIe	WRKYGQK	C-X_5_-C-X_23_-HXH	AtWRKY65	AT1G29280.1	
DcWRKY41	336	III	WRKYGQK	C-X_7_-C-X_23_-HTC	AtWRKY41	AT4G11070.1	
DcWRKY42	300	IIe	WRKYGQK	C-X_5_-C-X_23_-HXH	AtWRKY22	AT4G01250.1	drought, salt
DcWRKY43	306	IIc	WRKYGQK	C-X_4_-C-X_23_-HXH	AtWRKY71	AT1G29860.1	
DcWRKY44	187	IIa	WRKYGQK	C-X_5_-C-X_23_-HXH	AtWRKY40	AT1G80840.1	drought, salt
DcWRKY45	278	IIa	WRKYGQK	C-X_5_-C-X_23_-HXH	AtWRKY71	AT1G80840.1	drought, salt
DcWRKY46	337	I	WRKYGQK	C-X_4_-C-X_22_-HXH (N)/C-X_4_-C-X_23_-HXH (C)	AtWRKY33	AT2G38470.1	drought, salt
DcWRKY47	216	III	WRKYGQK	C-X_7_-C-X_23_-HTC	AtWRKY70	AT3G56400.1	
DcWRKY48	198	IIc	WRKYGQK	C-X_4_-C-X_23_-HXH	AtWRKY43	AT2G46130.1	
DcWRKY49	442	IIb	WRKYGQK	C-X_5_-C-X_23_-HXH	AtWRKY72	AT5G15130.1	
DcWRKY50	302	IIb	WRKYGQK	C-X_5_-C-X_23_-HXH	AtWRKY72	AT5G15130.1	
DcWRKY51	539	IIb	WRKYGQK	C-X_5_-C-X_23_-HXH	AtWRKY61	AT1G18860.1	
DcWRKY52	236	IIe	WRKYGQK	C-X_5_-C-X_23_-HXH	AtWRKY69	AT3G58710.1	
DcWRKY53	227	IIc	WRKYGQK	C-X_7_-C-X_23_-HTC	AtWRKY28	AT4G18170.1	cold, drought, salt
DcWRKY54	287	IIc	WRKYGQK	C-X_5_-C-X_23_-HXH	AtWRKY57	AT1G69310.2	
DcWRKY55	313	IIb	WRKYGQK	C-X_5_-C-X_23_-HXH	AtWRKY6	AT1G62300.1	cold, drought, salt
DcWRKY56	442	IIe	WRKYGQK	C-X_5_-C-X_23_-HXH	AtWRKY35	AT2G34830.1	
DcWRKY57	287	IIc	WRKYGQK	C-X_5_-C-X_23_-HXH	AtWRKY57	AT1G69310.2	
DcWRKY58	313	IId	WRKYGQK	C-X_5_-C-X_23_-HXH	AtWRKY15	AT2G23320.1	drought, salt
DcWRKY59	330	I	WRKYGQK	C-X_4_-C-X_23_-HXH	AtWRKY33	AT2G38470.1	drought, salt
DcWRKY60	324	IId	WRKYGQK	C-X_5_-C-X_23_-HXH	AtWRKY7	AT4G24240.1	salt
DcWRKY61	241	III	WRKYGQK	C-X_7_-C-X_23_-HTC	AtWRKY55	AT2G40740.1	
DcWRKY62	250	IIe	WRKYGQK	C-X_5_-C-X_23_-HXH	AtWRKY35	AT2G34830.1	
DcWRKY63	172	IIe	WRKYGQK	C-X_5_-C-X_23_-HXH	AtWRKY65	AT1G29280.1	
DcWRKY64	555	IIb	WRKYGQK	C-X_5_-C-X_23_-HXH	AtWRKY6	AT1G62300.1	cold, drought, salt
DcWRKY65	560	I	WRKYGQK	C-X_4_-C-X_22_-HXH (N)/C-X_4_-C-X_23_-HXH (C)	AtWRKY20	AT4G26640.2	
DcWRKY66	248	IIe	WRKYGQK	C-X_5_-C-X_23_-HXH	AtWRKY65	AT1G29280.1	
DcWRKY67	514	I	WRKYGQK	C-X_4_-C-X_22_-HXH (N)/C-X_4_-C-X_23_-HXH (C)	AtWRKY4	AT1G13960.1	drought, salt
DcWRKY68	507	I	WRKYGQK	C-X_4_-C-X_22_-HXH (N)/C-X_4_-C-X_23_-HXH (C)	AtWRKY33	AT2G38470.1	drought, salt
DcWRKY69	343	III	WRKYGQK	C-X_7_-C-X_23_-HTC	AtWRKY70	AT3G56400.1	
DcWRKY70	698	I	WRKYGQK	C-X_4_-C-X_22_-HXH (N)/C-X_4_-C-X_23_-HXH (C)	AtWRKY2	AT5G56270.1	heat
DcWRKY71	332	IIc	WRKYGQK	C-X_4_-C-X_23_-HXH	AtWRKY23	AT2G47260.1	
DcWRKY72	189	IIc	WRKYGKK	C-X_4_-C-X_23_-HXH	AtWRKY51	AT5G64810.1	cold
DcWRKY73	250	IIe	WRKYGQK	C-X_5_-C-X_23_-HXH	AtWRKY65	AT1G29280.1	
DcWRKY74	318	IIe	WRKYGQK	C-X_5_-C-X_23_-HXH	AtWRKY22	AT4G01250.1	drought, salt
DcWRKY75	286	IIe	WRKYGQK	C-X_5_-C-X_23_-HXH	AtWRKY65	AT1G29280.1	
DcWRKY76	254	IId	WKKYDHK	C-X_5_-C-X_23_-HXH	AtWRKY7	AT4G24240.1	salt
DcWRKY77	233	IIe	WRKYGQK	C-X_5_-C-X_23_-HXH	AtWRKY65	AT1G29280.1	
DcWRKY78	260	IIe	WRKYGQK	C-X_5_-C-X_23_-HXH	AtWRKY69	AT3G58710.2	
DcWRKY79	302	IIb	WRKYGQK	C-X_5_-C-X_22_-HXH	AtWRKY72	AT5G15130.1	
DcWRKY80	278	IIa	WRKYGQK	C-X_5_-C-X_23_-HXH	AtWRKY40	AT1G80840.1	drought, salt
DcWRKY81	324	I	WRKYGQK	C-X_4_-C-X_22_-HXH (N)/C-X_4_-C-X_23_-HXH (C)	AtWRKY3	AT2G03340.1	
DcWRKY82	287	IIc	WRKYGQK	C-X_4_-C-X_22_-HXH	AtWRKY48	AT5G49520.1	drought, salt
DcWRKY83	175	IIe	WRKYGQK	C-X_5_-C-X_23_-HXH	AtWRKY69	AT3G58710.1	
DcWRKY84	311	IIe	WRKYGQK	C-X_5_-C-X_24_-HXH	AtWRKY22	AT4G01250.1	drought, salt
DcWRKY85	310	IIb	WRKYGQK	C-X_5_-C-X_23_-HXH	AtWRKY72	AT5G15130.1	
DcWRKY86	338	III	WRKYGQK	C-X_7_-C-X_23_-HTC	AtWRKY41	AT4G23810.1	
DcWRKY87	276	IId	WRKYGQK	C-X_5_-C-X_23_-HXH	AtWRKY11	AT4G31550.2	drought
DcWRKY88	297	IId	WKKYDQK	C-X_5_-C-X_23_-HXH	AtWRKY11	AT4G31550.1	drought
DcWRKY89	865	IIb	WRKYGQK	C-X_5_-C-X_23_-HXH	AtWRKY61	AT1G18860.1	
DcWRKY90	282	IIe	WRKYGQK	C-X_5_-C-X_23_-HXH	AtWRKY22	AT4G01250.1	drought, salt
DcWRKY91	287	IIc	WRKYGQK	C-X_4_-C-X_23_-HXH	AtWRKY57	AT1G69310.2	
DcWRKY92	294	III	WRKYGQK	C-X_7_-C-X_23_-HXH	AtWRKY49	AT5G43290.1	
DcWRKY93	539	I	WRKYGQK	C-X_4_-C-X_22_-HXH (N)/C-X_4_-C-X_23_-HXH (C)	AtWRKY1	AT2G04880.2	
DcWRKY94	480	IIb	WRKYGQK	C-X_5_-C-X_23_-HXH	AtWRKY9	AT1G68150.1	
DcWRKY95	184	IIc	WRKYGQK	C-X_4_-C-X_23_-HXH	AtWRKY75	AT5G13080.1	salt

**Table 2:** Numbers of paralogous, orthologous, and coorthologous gene pairs among nine plant species.

	Cre	Ppa	Smo	Pab	Ath	Dca	Vvi	Mdo	Osa
Cre	1								
Ppa	0/0	67							
Smo	2/2	8/37	6						
Pab	0/0	7/26	7/12	237					
Ath	0/0	6/12	8/7	11/5	26				
Dca	0/0	8/25	10/14	10/12	28/23	56			
Vvi	0/0	5/12	7/5	15/40	31/16	36/38	7		
Mdo	0/0	4/29	12/22	14/29	34/52	46/97	48/72	102	
Osa	0/0	2/5	8/11	8/7	18/8	21/19	19/11	19/29	41

The numbers on the diagonal represent the paralogous genes of each species, the numbers located before “/” represent the orthologous genes, and the numbers located after “/” represent the coorthologous genes. Cre: *C. reinhardtii*; Ppa: *P. patens*; Smo: *S. moellendorffii*; Pab: *P. abies*; Ath: *A. thaliana*; Dca: *D. carota*; Vvi: *Vitis vinifera*; Mdo: *Malus domestica*; Osa: *O. sativa*.
