# Supplementary material for: Genomic identification of WRKY transcription factors in carrot (Daucus carota) and analysis of evolution and homologous groups for plants
Source: Sci Rep. 2016 Mar 15;6:23101. doi: 10.1038/srep23101 (PMC4792144; doi:10.1038/srep23101)
Supplement: Supplementary Information [file srep23101-s1.doc]

**Genomic identification of WRKY transcription factors in carrot (*Daucus carota*) and analysis of evolution and homologous groups for plants**

Meng-Yao Li, Zhi-Sheng Xu, Chang Tian, Ying Huang, Feng Wang, Ai-Sheng Xiong*

State Key Laboratory of Crop Genetics and Germplasm Enhancement, College of Horticulture, Nanjing Agricultural University, 1 Weigang, Nanjing 210095, China

*Please address all correspondence to: A.S. Xiong ([xiongaisheng@njau.edu.cn](mailto:Xiongaisheng@njau.edu.cn))

*-------------------------------*

Dr. Ai-Sheng Xiong

Professor

State Key Laboratory of Crop Genetics and Germplasm Enhancement,

College of Horticulture,

Nanjing Agricultural University,

Nanjing, 210095, China

Tel: 86 25 84396790

Fax: 86 25 84396790

Email: [xiongaisheng@njau.edu.cn](mailto:Xiongaisheng@njau.edu.cn)

**Supplemental Tables**

| **Table S1. Physical and chemical characterization of WRKY transcription factors.　‘–’ was any other location without chloroplast, mitochondrion, secretory pathwayin cell** | | | | | | | | | | | | |
| --- | --- | --- | --- | --- | --- | --- | --- | --- | --- | --- | --- | --- |
| **Group** | **Annotation ID** | **Gene name** | **Number of amino acid** | **pI** | **Percentage of Positive amino acid /%** | **Percentage of** **Negative amino acid /%** | **Percentage of Aliphatic amino acid /%** | **Percentage of Aromatics amino acid /%** | **Aliphatic index** | **Instability index** | **Grand average of hydropathicity** | **Sublocation (WoLF/TargetP)** |
| I | Dck03736 | DcWRKY6 | 472 | 9.09 | 18 | 11 | 17 | 5 | 65.12 | 45.17 | -0.871 | chloroplast/  chloroplast |
| I | Dck10344 | DcWRKY15 | 514 | 7.25 | 14 | 11 | 14 | 7 | 55.04 | 63.32 | -0.837 | nuclear/_ |
| I | Dck17526 | DcWRKY24 | 507 | 5.99 | 12 | 11 | 11 | 10 | 42.92 | 54.48 | -1.000 | nuclear/_ |
| I | Dck17800 | DcWRKY26 | 324 | 9.06 | 20 | 13 | 16 | 6 | 59.20 | 48.26 | -1.055 | nuclear/_ |
| I | Dck18473 | DcWRKY27 | 691 | 5.82 | 13 | 12 | 12 | 7 | 49.99 | 60.20 | -0.854 | nuclear/_ |
| I | Dck18493 | DcWRKY28 | 298 | 9.44 | 16 | 9 | 14 | 8 | 55.60 | 37.29 | -0.856 | chloroplast/_ |
| I | Dck18704 | DcWRKY30 | 551 | 6.9 | 12 | 10 | 12 | 8 | 46.73 | 56.78 | -0.995 | nuclear/  chloroplast |
| I | Dck22994 | DcWRKY33 | 531 | 5.94 | 15 | 14 | 18 | 3 | 64.16 | 52.19 | -0.097 | nuclear/_ |
| I | Dck23273 | DcWRKY34 | 429 | 6.34 | 14 | 12 | 15 | 7 | 57.90 | 47.81 | -0.855 | nuclear/mitochondrial |
| I | Dck30453 | DcWRKY46 | 337 | 8.94 | 16 | 11 | 14 | 7 | 52.61 | 44.92 | -1.002 | nuclear/_ |
| I | Dck38089 | DcWRKY59 | 330 | 9.13 | 16 | 11 | 14 | 7 | 53.73 | 45.77 | -0.990 | nuclear/_ |
| I | Dck53864 | DcWRKY65 | 560 | 6.53 | 13 | 11 | 15 | 7 | 56.39 | 50.20 | -0.764 | nuclear/  chloroplast |
| I | Dck55501 | DcWRKY67 | 514 | 6.96 | 14 | 11 | 14 | 7 | 54.28 | 63.72 | -0.839 | nuclear/_ |
| I | Dck55874 | DcWRKY68 | 507 | 5.99 | 12 | 11 | 11 | 10 | 42.92 | 54.48 | -0.100 | nuclear/_ |
| I | Dck56875 | DcWRKY70 | 698 | 5.61 | 12 | 12 | 15 | 7 | 60.92 | 51.54 | -0.629 | nuclear/_ |
| I | Dck66102 | DcWRKY81 | 324 | 9.06 | 20 | 13 | 16 | 6 | 59.81 | 49.32 | -1.046 | nuclear/_ |
| I | Dck77188 | DcWRKY93 | 539 | 5.79 | 15 | 14 | 19 | 3 | 68.83 | 52.35 | -0.835 | nuclear/_ |
|  |  | Average | 478 | 7.28 | 15 | 12 | 15 | 7 | 55.66 | 51.64 | -0.801 |  |
| II a | Dck20393 | DcWRKY31 | 343 | 8.22 | 14 | 11 | 16 | 6 | 65.16 | 42.93 | -0.720 | nuclear/_ |
| II a | Dck27455 | DcWRKY39 | 179 | 9.6 | 16 | 9 | 15 | 7 | 61.68 | 50.01 | -0.685 | nuclear/_ |
| II a | Dck30166 | DcWRKY44 | 187 | 8.91 | 17 | 12 | 18 | 7 | 68.82 | 51.99 | -0.640 | nuclear/_ |
| II a | Dck30167 | DcWRKY45 | 278 | 6.4 | 15 | 13 | 19 | 6 | 69.71 | 46.68 | -0.736 | nuclear/_ |
| II a | Dck65890 | DcWRKY80 | 278 | 6.4 | 15 | 13 | 19 | 6 | 69.71 | 46.68 | -0.736 | nuclear/_ |
|  |  | Average | 253 | 7.9 | 15 | 12 | 17 | 6 | 67.02 | 47.66 | -0.703 |  |
| II b | Dck00350 | DcWRKY1 | 598 | 5.36 | 12 | 12 | 14 | 4 | 59.46 | 49.87 | -0.797 | nuclear/  chloroplast |
| II b | Dck01527 | DcWRKY3 | 566 | 6.5 | 11 | 9 | 14 | 5 | 61.64 | 54.28 | -0.646 | nuclear/_ |
| II b | Dck01529 | DcWRKY4 | 436 | 6.6 | 12 | 10 | 15 | 5 | 63.83 | 55.04 | -0.616 | nuclear/_ |
| II b | Dck09790 | DcWRKY13 | 520 | 5.57 | 10 | 10 | 15 | 4 | 64.44 | 44.73 | -0.647 | nuclear/_ |
| II b | Dck17073 | DcWRKY23 | 522 | 6.7 | 13 | 9 | 15 | 4 | 62.13 | 56.56 | -0.663 | nuclear/_ |
| II b | Dck17598 | DcWRKY25 | 532 | 6.74 | 13 | 9 | 15 | 4 | 61.50 | 55.97 | -0.679 | nuclear/_ |
| II b | Dck31935 | DcWRKY49 | 442 | 8.12 | 12 | 9 | 15 | 6 | 61.18 | 51.74 | -0.692 | nuclear/_ |
| II b | Dck31936 | DcWRKY50 | 302 | 6.18 | 21 | 18 | 14 | 4 | 54.57 | 54.42 | -1.136 | nuclear/_ |
| II b | Dck32062 | DcWRKY51 | 539 | 6.22 | 13 | 11 | 13 | 6 | 54.56 | 59.16 | -0.802 | nuclear/  chloroplast |
| II b | Dck34830 | DcWRKY55 | 313 | 5.67 | 11 | 10 | 15 | 4 | 63.52 | 45.76 | -0.676 | nuclear/_ |
| II b | Dck52415 | DcWRKY64 | 555 | 5.9 | 12 | 10 | 15 | 5 | 62.29 | 41.87 | -0.703 | nuclear/_ |
| II b | Dck64650 | DcWRKY79 | 302 | 6.22 | 21 | 18 | 14 | 4 | 54.57 | 54.46 | -1.138 | nuclear/_ |
| II b | Dck70045 | DcWRKY85 | 310 | 8.62 | 15 | 12 | 15 | 6 | 63.29 | 49.94 | -0.659 | nuclear/_ |
| II b | Dck71859 | DcWRKY89 | 865 | 6.93 | 11 | 10 | 20 | 7 | 84.45 | 46.15 | -0.154 | nuclear/_ |
| II b | Dck77521 | DcWRKY94 | 480 | 6.09 | 15 | 14 | 16 | 5 | 64.62 | 52.43 | -0.878 | nuclear/_ |
|  |  | Average | 485 | 6.49 | 13 | 11 | 15 | 5 | 62.40 | 51.49 | -0.726 |  |
| II c | Dck03731 | DcWRKY5 | 287 | 6.1 | 14 | 13 | 16 | 7 | 63.52 | 69.89 | -0.787 | nuclear/_ |
| II c | Dck08974 | DcWRKY11 | 175 | 9.21 | 20 | 12 | 14 | 9 | 52.34 | 48.35 | -1.070 | nuclear/_ |
| II c | Dck11107 | DcWRKY17 | 242 | 8.42 | 13 | 9 | 17 | 9 | 64.01 | 48.74 | -0.689 | nuclear/  secretory pathway |
| II c | Dck12342 | DcWRKY19 | 204 | 6.19 | 16 | 13 | 15 | 10 | 59.26 | 35.46 | -0.831 | nuclear/_ |
| II c | Dck16336 | DcWRKY21 | 163 | 9.34 | 19 | 13 | 15 | 10 | 57.42 | 37.99 | -0.817 | mitochondrio/  mitochondrio |
| II c | Dck16337 | DcWRKY22 | 157 | 5.78 | 16 | 16 | 13 | 11 | 52.80 | 43.65 | -0.804 | nuclear/_ |
| II c | Dck25338 | DcWRKY37 | 250 | 8.94 | 17 | 10 | 15 | 8 | 56.12 | 59.95 | -0.969 | nuclear/_ |
| II c | Dck25547 | DcWRKY38 | 305 | 7.2 | 15 | 11 | 13 | 9 | 50.85 | 55.24 | -0.961 | nuclear/_ |
| II c | Dck29697 | DcWRKY43 | 306 | 6.27 | 16 | 13 | 13 | 9 | 49.71 | 52.38 | -0.825 | nuclear/_ |
| II c | Dck30999 | DcWRKY48 | 198 | 8.31 | 17 | 12 | 14 | 9 | 48.69 | 48.23 | -0.933 | nuclear/_ |
| II c | Dck32735 | DcWRKY53 | 227 | 6.51 | 17 | 14 | 15 | 11 | 60.62 | 44.55 | -0.795 | nuclear/_ |
| II c | Dck34690 | DcWRKY54 | 287 | 6.06 | 14 | 13 | 13 | 9 | 52.40 | 51.45 | -0.858 | nuclear/_ |
| II c | Dck36498 | DcWRKY57 | 287 | 6.06 | 14 | 13 | 13 | 9 | 52.40 | 51.45 | -0.858 | nuclear/_ |
| II c | Dck57113 | DcWRKY71 | 332 | 7.66 | 12 | 10 | 16 | 7 | 58.64 | 58.59 | -0.780 | nuclear/_ |
| II c | Dck57155 | DcWRKY72 | 189 | 6.58 | 16 | 13 | 17 | 8 | 65.98 | 43.76 | -0.726 | nuclear/_ |
| II c | Dck66934 | DcWRKY82 | 287 | 6.6 | 14 | 12 | 16 | 7 | 62.16 | 66.85 | -0.782 | nuclear/_ |
| II c | Dck76502 | DcWRKY91 | 287 | 6.06 | 14 | 13 | 13 | 9 | 52.40 | 51.45 | -0.858 | nuclear/_ |
| II c | Dck78428 | DcWRKY95 | 184 | 9.42 | 16 | 10 | 11 | 10 | 43.42 | 41.38 | -1.025 | nuclear/  chloroplast |
|  |  | Average | 245 | 7.13 | 15 | 12 | 15 | 9 | 56.25 | 49.96 | -0.843 |  |
| II d | Dck06192 | DcWRKY9 | 101 | 9.52 | 22 | 11 | 16 | 6 | 57.82 | 35.58 | -0.985 | nuclear/_ |
| II d | Dck10828 | DcWRKY16 | 338 | 9.66 | 17 | 7 | 16 | 6 | 63.49 | 53.31 | -0.784 | nuclear/_ |
| II d | Dck14242 | DcWRKY20 | 346 | 9.82 | 17 | 8 | 18 | 6 | 71.01 | 54.66 | -0.629 | nuclear/_ |
| II d | Dck24488 | DcWRKY35 | 248 | 9.78 | 21 | 10 | 16 | 6 | 62.90 | 60.02 | -0.856 | nuclear/_ |
| II d | Dck37435 | DcWRKY58 | 313 | 9.8 | 18 | 8 | 17 | 6 | 65.40 | 39.66 | -0.584 | nuclear/_ |
| II d | Dck40716 | DcWRKY60 | 324 | 9.46 | 17 | 10 | 16 | 6 | 62.28 | 57.75 | -0.683 | nuclear/_ |
| II d | Dck61677 | DcWRKY76 | 254 | 10.04 | 21 | 8 | 16 | 7 | 62.99 | 59.87 | -0.748 | nuclear/_ |
| II d | Dck71179 | DcWRKY87 | 276 | 9.31 | 20 | 11 | 18 | 5 | 69.20 | 51.91 | -0.668 | chloroplast/_ |
| II d | Dck71515 | DcWRKY88 | 297 | 9.72 | 21 | 10 | 19 | 4 | 68.86 | 61.99 | -0.690 | nuclear/_ |
|  |  | Average | 277 | 9.68 | 19 | 9 | 17 | 6 | 64.88 | 52.75 | -0.736 |  |
| II e | Dck09010 | DcWRKY12 | 332 | 5.94 | 13 | 12 | 12 | 7 | 50.87 | 62.25 | -0.796 | nuclear/_ |
| II e | Dck10146 | DcWRKY14 | 305 | 5.06 | 15 | 17 | 17 | 10 | 66.13 | 55.99 | -0.772 | nuclear/_ |
| II e | Dck11936 | DcWRKY18 | 311 | 5.14 | 14 | 16 | 13 | 10 | 50.51 | 54.18 | -0.873 | nuclear/_ |
| II e | Dck18548 | DcWRKY29 | 233 | 5.04 | 14 | 16 | 16 | 9 | 61.46 | 58.02 | -0.594 | nuclear/_ |
| II e | Dck25249 | DcWRKY36 | 397 | 6.21 | 14 | 12 | 16 | 7 | 62.67 | 45.65 | -0.737 | nuclear/_ |
| II e | Dck28185 | DcWRKY40 | 219 | 5.7 | 15 | 14 | 14 | 9 | 55.66 | 47.65 | -0.743 | nuclear/_ |
| II e | Dck29003 | DcWRKY42 | 300 | 5.25 | 14 | 15 | 16 | 10 | 60.43 | 40.82 | -0.632 | nuclear/_ |
| II e | Dck32456 | DcWRKY52 | 236 | 5.5 | 13 | 13 | 15 | 8 | 54.49 | 44.12 | -0.780 | nuclear/_ |
| II e | Dck35317 | DcWRKY56 | 442 | 5.28 | 11 | 12 | 14 | 7 | 55.61 | 48.32 | -0.767 | nuclear/_ |
| II e | Dck42367 | DcWRKY62 | 250 | 5.55 | 13 | 14 | 13 | 8 | 51.48 | 50.52 | -0.955 | nuclear/_ |
| II e | Dck48048 | DcWRKY63 | 172 | 6.15 | 21 | 20 | 12 | 5 | 45.87 | 62.81 | -1.455 | nuclear/_ |
| II e | Dck53950 | DcWRKY66 | 248 | 4.97 | 16 | 20 | 14 | 8 | 55.44 | 64.77 | -1.110 | nuclear/_ |
| II e | Dck57306 | DcWRKY73 | 250 | 5.38 | 14 | 15 | 16 | 9 | 59.24 | 61.19 | -0.672 | nuclear/_ |
| II e | Dck57752 | DcWRKY74 | 318 | 4.61 | 11 | 16 | 133 | 10 | 51.54 | 51.32 | -0.909 | nuclear/_ |
| II e | Dck60437 | DcWRKY75 | 286 | 5.38 | 13 | 13 | 14 | 7 | 53.88 | 54.71 | -0.893 | nuclear/_ |
| II e | Dck62670 | DcWRKY77 | 233 | 5.67 | 17 | 17 | 16 | 11 | 55.19 | 37.39 | -0.849 | nuclear/_ |
| II e | Dck62677 | DcWRKY78 | 260 | 5.69 | 17 | 17 | 15 | 9 | 52.46 | 42.35 | -0.908 | nuclear/_ |
| II e | Dck67485 | DcWRKY83 | 175 | 5.72 | 21 | 21 | 13 | 5 | 47.31 | 66.08 | -1.455 | nuclear/_ |
| II e | Dck68077 | DcWRKY84 | 311 | 5.14 | 14 | 16 | 13 | 10 | 51.77 | 56.04 | -0.848 | nuclear/_ |
| II e | Dck72993 | DcWRKY90 | 282 | 5.08 | 14 | 16 | 18 | 9 | 68.44 | 46.42 | -0.743 | nuclear/_ |
|  |  | Average | 278 | 5.42 | 15 | 16 | 21 | 8 | 55.52 | 52.53 | -0.875 |  |
| III | Dck01131 | DcWRKY2 | 318 | 6.32 | 14 | 13 | 17 | 8 | 66.26 | 52.36 | -0.715 | nuclear/_ |
| III | Dck04992 | DcWRKY7 | 351 | 5.14 | 12 | 24 | 17 | 8 | 66.70 | 49.86 | -0.634 | nuclear/_ |
| III | Dck06178 | DcWRKY8 | 332 | 6.14 | 14 | 11 | 15 | 9 | 61.11 | 53.81 | -0.782 | nuclear/_ |
| III | Dck06911 | DcWRKY10 | 294 | 8.38 | 15 | 11 | 16 | 7 | 6.02 | 65.04 | -0.743 | nuclear/  chloroplast |
| III | Dck22214 | DcWRKY32 | 390 | 7.22 | 14 | 11 | 16 | 10 | 59.26 | 52.54 | -0.754 | nuclear/_ |
| III | Dck28407 | DcWRKY41 | 336 | 5.62 | 13 | 12 | 15 | 10 | 61.88 | 53.08 | -0.672 | nuclear/_ |
| III | Dck30783 | DcWRKY47 | 216 | 8.21 | 17 | 13 | 15 | 7 | 57.31 | 62.47 | -0.811 | nuclear/_ |
| III | Dck42023 | DcWRKY61 | 241 | 8.39 | 16 | 12 | 14 | 7 | 53.03 | 62.27 | -0.957 | nuclear/_ |
| III | Dck56774 | DcWRKY69 | 343 | 6.59 | 14 | 11 | 16 | 7 | 61.11 | 52.71 | -0.660 | nuclear/_ |
| III | Dck70477 | DcWRKY86 | 338 | 5.34 | 14 | 14 | 15 | 9 | 60.06 | 51.35 | -0.764 | nuclear/_ |
| III | Dck77037 | DcWRKY92 | 294 | 4.71 | 11 | 15 | 19 | 8 | 65.95 | 39.88 | -0.646 | nuclear/_ |
|  |  | Average | 316 | 6.74 | 14 | 13 | 16 | 8 | 55.27 | 55.55 | -0.749 |  |
| **Total** |  | Average | 333 | 7.24 | 15 | 12 | 16 | 7 | 59.57 | 51.65 | -0.776 |  |

**Table S2. Sequences of primers used in qRT-PCR, subcellular localization, and yeast two-hybrid.**

| **Gene ID** | **Purpose** | **Forward primer (5'→3')** | **Reverse primer (5'→3')** |
| --- | --- | --- | --- |
| *DcWRKY1* | For qRT-PCR | AGGCACGATGGCTGACACTGT | GTGGAAGTTGTGGCATTGGCATTG |
| *DcWRKY2* | For qRT-PCR | AACAAGGAAGAGCCGAGTGATGA | CCAGCACTGGTATCCACACGATT |
| *DcWRKY5* | For qRT-PCR | TACAACCTGCGGTGTCCTGAAGA | CTGAATGCTCTGCTGCTGGAGTC |
| *DcWRKY6* | For qRT-PCR | GCGTGGTCCAGAGTTCCAATGAT | TTGACTGGCGGCTTGTGATGATT |
| *DcWRKY8* | For qRT-PCR | ACAACTCAGTTCCACCAGCAACAC | AGCACATCGTTCGTTGGTTCTTGA |
| *DcWRKY10* | For qRT-PCR | CCTGGTGATGCTAGTCCAAGAAGT | TGTTCACTCCATTGTGTCATTGCC |
| *DcWRKY11* | For qRT-PCR | TCAATCTCCAGCTTCACACGATCA | TTCCGCTGCTTGCTCTTATCATCA |
| *DcWRKY18* | For qRT-PCR | CTGATTGCTGGGCTTGGCGTAA | CGTGGTTGTGTTCGGCTGAGTT |
| *DcWRKY23* | For qRT-PCR | CGTCCTCCGTGGTCGATGAAGA | CGCCAATCTGCTGTTCTGGTGTT |
| *DcWRKY24* | For qRT-PCR | ACCAGTGCTCCCACGGCTAT | TCGTCCTTGGCTGTGGAGAACT |
| *DcWRKY27* | For qRT-PCR | CCATGATGTTCCTGCTGCTCGTAA | GGTCCCATTTGCTGCCTTCCA |
| *DcWRKY28* | For qRT-PCR | ATGGAGTCTGCGGCATCTCATC | GCTGTGCTGTGGCTGCTGTT |
| *DcWRKY30* | For qRT-PCR | GGAGATGTCAACAACGCTGTCAGT | TGGCAGAATAGGAGAGTCCAAGAGT |
| *DcWRKY31* | For qRT-PCR | AATGCCAACCCTCGTCAACTCTTT | TCACTCTGTTCAACTCCTCAACCA |
| *DcWRKY39* | For qRT-PCR | TCGAAGTGCTGCAATAGCCTCA | GCTGCGAGTGCTGCTTTGAAA |
| *DcWRKY45* | For qRT-PCR | GCTCGTCTTCGCCAAGAGAACC | ACATCTTTGTTGTGCCCTGCTGAC |
| *DcWRKY47* | For qRT-PCR | GGTCAAGGATCGTAGAGGATGCT | GGTACGGTACATTGGTGCTGGAT |
| *DcWRKY58* | For qRT-PCR | CGTCGTCGTTTAAGCGGAAGTGT | TAGGACAATGGCACCTCCCAGAAG |
| *DcWRKY64* | For qRT-PCR | GCAATGGCAATGGCATCCTCAAC | AATGGAGCGGAGGCGGAGAT |
| *DcWRKY67* | For qRT-PCR | GCTGCCTTCTCTGTTACCTGCC | CGCTCGCTTTGACCTGCTTCT |
| *DcWRKY68* | For qRT-PCR | ACCAGTGCTCCCACGGCTAT | TCGTCCTTGGCTGTGGAGAACT |
| *DcWRKY69* | For qRT-PCR | CCAGCACCAATGTACCGTACCA | TGCGAGTTGAAGAAGTGGAGTGG |
| *DcWRKY71* | For qRT-PCR | CCGGAGCCCAGTTCAGAATCAA | TGTGCTGCTGCTGTTGTTCTTG |
| *DcWRKY74* | For qRT-PCR | ACCTCCGCCTCTGTCGTTGAT | TTGGACTGTGGCTCCTGTTGTG |
| *DcWRKY80* | For qRT-PCR | ACGGAGAACGAGAGGCTGACTA | CCTTCACCACCAGGCTCTTGTT |
| *DcWRKY88* | For qRT-PCR | CGTTGCCGACTTCTCACAGGAA | AGTGACAACCACGAGAGCCAGA |
| *DcWRKY90* | For qRT-PCR | ATGATCCTCCAGGACCGACACT | CGCCAAGCCCATAAATCCGAAG |
| *DcWRKY95* | For qRT-PCR | TCAGACGAGGAGCCAAGTGGAT | GTCACCACAACGCCTTCATCCT |
| *DcTUB* | For qRT-PCR | CGGTATTGTGTTGGACTCTGGTGAT | CAGCAAGGTCAAGACGGAGTATGG |
| *DcWRKY11* | For subcellular localization | ACACGGGGGACTCTTGACCATGGATGTTTCATCAATCTCCAGCTTC | TTACTAGTCAGATCTACCATGGATTTGTTGTATGGTAAATTTGCATC |
| *DcWRKY45* | For subcellular localization | ACACGGGGGACTCTTGACCATGGATGGACTCCCCTACGGTTAA | TTACTAGTCAGATCTACCATGGCCATCCATCCATGAGATCATA |
| *DcWRKY80* | For subcellular localization | ACACGGGGGACTCTTGACCATGGATGGCTGTTGATCTTATAAATCTA | TTACTAGTCAGATCTACCATGGCAGCATATACCAACACCACTA |
| *DcWRKY20* | For yeast two-hybrid | ACGAGAATTCTGGCCATGGAGGCCGAATTCATG | CAGAGGATCCCGCTGCAGGTCGACGGATCCTCAC |
| *DcMAPK1* | For yeast two-hybrid | ACGAGAATTCCCATGGAGGCCAGTGAATTCAT | CAGAGGATCCAGCTCGAGCTCGATGGATCCTTAT |
| *DcMAPK4* | For yeast two-hybrid | ACGTGAATTCCCATGGAGGCCAGTGAATTCAT | CAGAGGATCCAGCTCGAGCTCGATGGATCCTTAA |
